# Fresh Properties and Sulfuric Acid Resistance of Sustainable Mortar Using Alkali-Activated GGBS/Fly Ash Binder

**DOI:** 10.3390/polym14030591

**Published:** 2022-02-01

**Authors:** Osama Ahmed Mohamed, Rania Al Khattab

**Affiliations:** College of Engineering, Abu Dhabi University, Abu Dhabi P.O. Box 59911, United Arab Emirates; raniaW178@yahoo.com

**Keywords:** alkali-activated fly ash and slag, concrete durability, sorptivity, alkaline activator ratio, decrease of CO_2_ emissions, sulphuric acid, compressive strength

## Abstract

In this study, sorptivity, setting time, resistance to sulfuric acid, and compressive strength of mortars that use alkali-activated GGBS and fly ash as binders, were evaluated experimentally. The activation of binders, was achieved at room temperature of 22 ± 2 °C using combinations of sodium silicates (Na_2_SiO_3_) and sodium hydroxide (NaOH) solutions in ratios of 1.5, 2.0, and 2.5. The parameters considered in terms of their effects on fresh and hardened properties include: NaOH molarity, activator ratio Na_2_SiO_3_/NaOH, mortar sample age, and relative amount of GGBS/fly ash in binder combination. Sorptivity, change in mass, and compressive strength were determined for mortar samples that were submerged in 10% sulfuric acid solution for 7 days, 28 days, and 90 days. The binder for mortar samples tested at each of the specified ages consisted of 100% GGBS (G100), 75%GGBS+25% fly ash (G75F25), or 50% GGBS + 50% fly ash (G50F50). The binder was activated using Na_2_SiO_3_ solution, combined with 10 M, 12 M, 14 M, or 16 M NaOH solution. It was found that sorptivity decreases with increase in curing age, for all activator ratios, concentrations, and relative amounts of GGBS/fly ash. Binder consisting of 75%GGBS + 25% fly ash with NaOH concentration of 12 M had the lowest sorptivity. Exposure of alkali-activated GGBS/fly ash mortar samples to sulfate attack did not cause loss in mass nor visible signs of damage/deterioration. All binder combinations experienced increase in compressive strength after curing in 10%sufluric acid solution, with the optimum G75F25 mix achieving a 28-day strength of 80.53 MPa when NaOH molarity is 10 M, which increased to 91.06 MPa after 90 days. Variation in concentration of NaOH didn’t cause significant change in the magnitudes of 28-day or 90-day compressive strengths of G50F50. However, despite slow dissolution of fly ash and immersion in 10% sulfuric acid solution, G50F50 developed 28-day compressive strength of 56.23 MPa and 90-day compressive of 86.73 MPa, which qualifies G50F50 as high strength mortar for practical purposes.

## 1. Introduction

Typical design considerations for suitability of concrete in a typical application include workability/flowability, setting time, durability, and compressive strength. These properties have been studied extensively for traditional concrete that uses ordinary Portland cement (OPC) as binder. The production of OPC has been associated with significant emission of CO_2_, a global problem that received significant attention in the past decades. Therefore, there is a renewed interest in studying alkali-activated natural or industrial byproducts as alternative binders to OPC. Studies have shown that long-term exposure of concrete to aggressive environment, such as freezing and thawing, or alkali-aggregate reaction may cause significant loss of strength and impair load-carrying capacity of structural elements [1]. The penetrability of concrete pore system is a measure of its performance in aggressive environment, and has therefore been linked to its durability and long-term resilience. In unsaturated concrete, the rate of water ingress is controlled to great extent by absorption due to capillary rise. Studies have shown that ingress of water carrying harmful chlorides into concrete may lead to corrosion of reinforcing steel bars and jeopardize the integrity of the structural system [2]. The conveyance of chloride-bearing water to reinforcing steel through concrete is related fundamentally to the connectivity of the pore system, in addition to pore structure and microcracks. Fly ash and ground-granulated blast furnace slag (GGBS), individually or combined have demonstrated ability to enhance mechanical properties and durability, when used as concrete binders to partially replace OPC [3,4]. 

A study by Collins and Sanjayan [5] showed that sorptivity of 63 mm × 98 mm concrete samples produced using alkali-activated GGBS binders, increases mildly with time when samples are left unsealed under lab conditions at 23 °C and 50% relative humidity (RH). On the other hand, sorptivity decreased continuously for all samples cured in water or sealed but left in lab conditions, for all ages from 1 day to 96 days. Zhang and Zong [6] found that water absorption of 100 mm × 175 mm OPC-based cylindrical concrete samples was higher when cured in the lab by direct exposure to air at 20 ± 3 °C and 65 ± 5% RH, compared to samples cured under water. On the other hand, water absorption was much less when the same concrete samples were air-cured at the same temperature (20 ± 3 °C), but at higher relative humidity of 90 ± 5%. However, air-cured samples developed higher compressive strength than moist-cured samples, especially at higher levels of RH which is consistent with the literature [7].

Water absorption of mortars in which fly ash precursor was activated using a combination of NaOH and Na_2_SiO_3_ was found to be affected by curing temperature as well as the activator ratio Na_2_SiO_3_/NaOH. Studies in which fly ash mortar samples were cured for 24 h initially at 100 °C, absorbed less water than those cured at 70 °C for all ratios of Na_2_SiO_3_/NaOH including 1.0, 1.5, 2.0, 2.5, and 3.0. However, samples cured at 100 °C experienced increase in rate of water absorption with increase in activator ratio. At the lower curing temperature of 70 °C, rate of water absorption decreased with increase in activator ratio [8].

Setting time and flowability are greatly enhanced with a balanced amount of fly ash in the binder mix, while GGBS enhances early strength development and carbonation resistance. Nedeljković et al. [9] indicated that increasing the content of GGBS in GGBS/fly ash binder mixes enhances carbonation resistance of alkali-activated mortar samples, regardless of curing conditions.

Calcium stearate (CaSt) in optimum dosages decreases sorptivity of alkali-activated slag compared to benchmark OPC mortar samples [10]. CaSt is hypothesized to enhance the pore system by decreasing pore connectivity/microcracks, and increasing entrained pores. The admixture also introduces a hydrophobic film on the pores that decreases water sorptivity. However, CaSt tends to decrease compressive strength of concrete. 

Key combinations of GGBS and fly ash that offered promising results in the literature are 50%GGBS+50% fly ash and 75%GGBS+25% fly ash, which are evaluated in this study, in addition to 100%GGBS mixes to serve as benchmark. Studies on alkali-activated GGBS as sole binder demonstrated significant benefits in terms of early strength development and durability of concrete, along with some shortcomings. Combining fly ash with GGBS as alkali-activated binders could alleviate the high shrinkage experienced by concrete/mortars when GGBS is used as sole binder [11]. 

When exposed to acid, early scaling and softening of OPC-based concrete has been observed due to decomposition of calcium hydroxide and formation of large amount of gypsum. As a result, long-term exposure of concrete to aggressive acid-rich environment, such as sewerage system, leads to severe degradation of strength [12,13]. The higher the content of OPC binder, the higher the mass loss due to exposure to sulfuric acid [14]. Theoretical studies by Ren et al. [15] predicted that, when subjected to sulfuric acid for 50-years, 52% to 60% higher loss in compressive strength may occur in alkali-activated slag/fly ash concrete compared to OPC-based concrete.

Zhang et al. [16] reported that exposure of alkali-activated mortars to sulfuric acid for 28-days has negligible effect on mineralogical properties when fly ash is the sole binder and formation of gypsum was limited to the outer surface of samples, without propagating to inner core of the samples. After immersion in sulfuric acid for 28-days, the layers of mortar samples prepared using blends of GGBS/fly ash affected by sulfate attack, experienced reduction in Al/Si ratio compared to layers or portions of the samples that were not affected by sulfate attack. In blends of GGBS and fly ash, the higher the percentage of GGBS the lower the effect of sulfuric acid, possibly due to the denser matrix and refined core structure formed by GGBS hydration products impeding progression of sulfuric acid.

Unlike mortars developed using alkali-activated GGBS as sole binder, exposure of fly ash-based mortar samples to 3% sulfuric acid for 90 days lead to severely compromised porosity. The increase in porosity system was attributed to the corrosive action of the acid and leaching of gypsum from the matrix [17]. Similarly, Bakharev [18] observed significant deterioration in strength along with loss of mass in geopolymer mortar samples prepared using class F fly ash and activated using sodium silicate and a mixture of sodium hydroxide and potassium hydroxide. Tests by Gu [19] showed that exposure of fly ash-based alkali-activated mortars to even 1% sulphuric acid for 492 days results in significant loss of mass and strength. XRD studies by Lee and Lee [20] on alkali-activated GGBS/fly ash showed that exposure to 10% sulfuric acid causes formation of gypsum due to decalcification of C-A-S-H. Contrary to studies reported earlier in this section, the formation of gypsum along with weight was more severe when GGBS content is increased from 0 to 50% at the expense of fly ash content in the total alkali-activated binder [21].

Fly ash-based geopolymer mortars subjected to MgSO_4_ solution for 10 years experienced deeper penetration of sulfates compared to OPC-based mortar samples. However, OPC-based samples experienced more swelling and cracking compared to fly ash samples, due to formation of gypsum and ettrignite when sulfate ions reacted with Ca(OH)_2_ and calcium aluminate hydrate [19]. 

It was reported that certain organic acids prevalent in wastewater and other environments, such as acetic acid has more damaging effect on highly alkaline cementitious materials, compared to strong acids such as sulfuric acids [22]. Other studies, however, described damage to cement-based materials due to acetic acid as intermediate while citric acid causes severe degradation to cement matrix [23]. In general, blended GGBS/fly ash concrete/mortar had better resistance to organic acids commonly found in agricultural and food effluents, such as acetic and lactic acids, compared to OPC-based concrete/mortars [22]. The enhanced resistance of alkali-activated concrete/mortar to acid-induced deterioration was attributed to reduced amount of vulnerable phases such as calcium hydroxide and ettrignite, compared to OPC-based concrete/mortar which contains those vulnerable phases. The vulnerability of alkali-activated concrete/mortar when fly ash is used as sole binder to sulfuric acid motivates the need to evaluate binders containing both fly ash and GGBS to benefit from the known strengths that each of the two material bring to the finished product. 

Concrete setting time is a critical property that affects workability and time needed to complete casting in professional practice, as well as time necessary to remove formwork in the case of cast-in-place (CIP) concrete structures. Study by Dehghani et al. [24] showed that in blended GGBS/fly ash mixes, the higher the percentage of GGBS the lower the initial and final setting times, and increasing the ratio of SiO_2_/Al_2_O_3_ decreases the setting time. In blends in which GGBS was decreased from 22% (78% fly ash) to 4% (96% fly ash), the initial setting time increased from approximately 35 min to 60 min, while the final setting time increased from approximately 55 min to nearly 100 min. This is attributed to abundance of calcium (in the form of CaO) in the GGBS precursor. Increasing fly ash content in the binder blend increases setting time due to the slow dissolution of fly ash and subsequent slow formation of polymerization products. Decrease in setting time in GGBS/fly ash blends where 70% of the binder is GGBS or the entire binder is GGBS is also documented by Jang et al. [25].

Wardhono et al. [26] studied compressive strength development of 50 mm cubic mortar samples cured by submerging under water at ambient temperature and tested after 3, 7, 14, and 28 days. Binders consisting of GGBS, or combination of fly ash class F and GGBS were activated using a mixture of 15M NaOH solution and sodium silicate solution. The activator solution was proportioned to achieve 15% Na_2_O content and a modulus (SiO_2_/Na_2_O) of 1.25. Mixes were created with various binder ratios of GGBS/fly ash. With the exception of the mix with 100% GGBS, all mixes increased in compressive strength starting from 3 days of curing to 28 days. After 28-days of moist curing at ambient temperature, the mortar samples with 50% GGBS and 50% fly ash exhibited the highest compressive strength. The 100% GGBS mix developed the highest compressive strength at the age of 3 days, but by the age of 7-days, the mix with 50%GGBS + 50% fly ash developed higher compressive strength. 

Many studies identified the main reaction product of alkali-activated GGBS-fly ash concrete to be chain-structured C-A-S-H type gel [27]. In addition to C-A-S-H, the presence of fly ash promotes formation of sodium aluminosilicate (N-A-S-H) which is more porous and less compact than C-A-S-H [28]. Uppalapati et al. [29] reported that in the early hours after mixing GGBS-fly ash binder combination, only C-A-S-H is present due to the fast hydration of GGBS with little or no N-A-S-H. This is because dissolution of fly ash takes longer time than hydration of GGBS. Nonetheless, Lee et al. [30] reported identifying C–N–A–S–H as reaction product in early-hours after mixing GGBS-fly ash mortar samples. Studies consistently reported that increasing GGBS content in GGBS-fly ash binders results in denser matrix of the reaction products.

In unsaturated concrete, the rate of water ingress is controlled to great extent by absorption due to capillary rise. The industrial by-products GGBS and fly ash are sustainable alternatives to OPC, that offer high potential of producing durable concrete for infrastructure projects. Curing method, temperature, and relative humidity affect strength development in concrete that uses OPC as binder. Concrete in which up to 90% of OPC was replaced by selected combinations of GGBS, fly ash, silica fume developed higher 28-day compressive strength when cured in air at 45 °C temperature and 70% RH, than similar OPC-based concrete cured under water in ambient laboratory conditions [31].

The objective of this study is to assess the effect of NaOH activator concentration (molarity), ratio of sodium silicate/sodium hydroxide (SS/SH), relative amount of GGBS and fly ash in binder mix, on the sorptivity setting time, and resistance to sulfuric acid attack of alkali-activated concrete.

## 2. Methodology

The experimental program was designed and implemented to evaluate initial/final setting times, sorptivity, and resistance to sulphuric acid of mortar samples considering the effect of alkalinity of the activator solution and the relative contents of GGBS/fly ash in the total binder content.

### 2.1. Material Properties 

Alkali-activated binder used in this study is GGBS or combination of GGBS and fly ash and their properties are shown in Table 1. The binders were supplied by RMB Ready Mix, Abu Dhabi, United Arab Emirates. The percentages of calcium oxide (CaO), Silicon dioxide (SiO_2_) + aluminum oxide (Al_2_O_3_) + iron oxide (Fe_2_O_3_), and Sulfur trioxide (SO_3_) are all consistent with ASTM C618 [32] class F fly ash. Sodium hydroxide flakes and sodium silicates solution used to activate the binder were supplied Dubichem, Dubai, United Arab Emirates.

### 2.2. Experimental Procedure—Sorptivity

The sorptivity of alkali-activated GGBS/fly ash mortar samples was evaluated using the methodology proposed by Hall [33] and adapted by ASTM C1585 [34]. The original test was developed to determine water absorption of hydraulic OPC. However, studies have shown that connected porosity, which controls transport properties of OPC-based and alkali-activated mortars are very similar [35,36]. The test measures the increase in the mass of a specimen resulting from absorption of water as a function of time through one surface of the specimen. The exposed surface of the specimen is immersed in water, and water ingress of unsaturated mortar specimens is dominated by capillary suction during initial contact with water. Measurement of sample weight and calculation of the rate of absorption offer a good indication of the continuity of pores within geopolymer mortar samples.

The activator solution was prepared by combining sodium hydroxide (NaOH) and sodium silicate solutions. NaOH solution is prepared by adding calculated amounts of sodium hydroxide flakes to clean water to develop various mixes having molarities of 10 M, 12 M, 14 M, and 16 M. After adding NaOH flakes to water, the solution is covered for two hours to cool down as the reaction is exothermic. Sodium silicate solution was then added to the sodium hydroxide solution in appropriate quantities to create various alkaline activator solutions with modulus n = Na_2_SiO_3_/NaOH of 1.5, 2.0, or 2.5. These alkaline activator mixtures (1.5, 2.0, and 2.5) were prepared for four sodium hydroxide mixes with molarities of 10 M, 12 M, 14 M, and 16 M producing a total of 12 alkaline activator mixes for each of the three binder types. The three binder types are 100% GGBS (G100), 75% GGBS + 25% fly ash (G75F25), and 50% GGBS + 50% fly ash (G50F50). For sorptivity tests, 100 mm (diameter) × 50 mm (length) mortar samples were prepared for each of the 36 mixes. 

Prior to preparing mortar samples, a polycarboxylic ether superplasticizer was added to the activator solution with adjusted dosage to enhance workability while limiting bleeding. The superplasticizer used is produced and marketed by BASF Corporation, Ludwigshafen, Germany, under the commercial name MasterGlenium SKY 504. The quantity of superplasticizer is 2.5% of the total binder content. The sand-to-binder-ratio is maintained at 2.75, while the activator solution-to-binder ratio was maintained at 0.55 for each of the 36 mixes. Table 2 shows the three binder groups based on GGBS and fly ash contents, and the corresponding 12 mixes for each of the three binder types, G100, G75F25, and G50F50.

**Figure 1:**
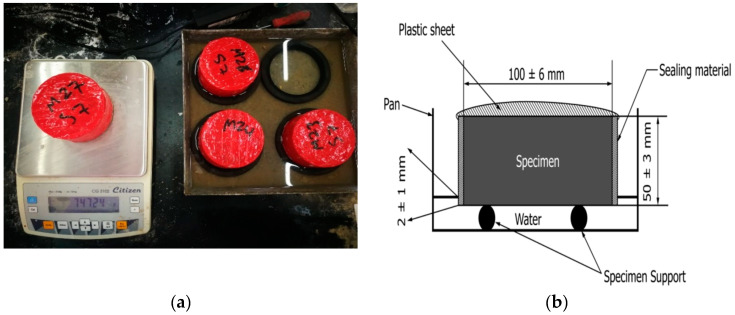
(**a**) Mortar samples immersed in water for the specified period of time, then weighed, (**b**) schematic for immersion level and sealing system/sides.

After demolding, the 100 mm × 50 mm samples were left in the laboratory until the sorptivity test date, which was done after 7 days, 28 days, and 90 days. Each sample was oven dried, then sealed with duct tape on each side, except the bottom, which will be in contact with water. Weight of sample was determined using 0.01g scale while dry, prior to placement on water. The samples were then placed so that 1 to 2 mm of the sample is immersed in water, as shown in Figure 1. The samples were then removed from the water box and weighed after 1, 5, 10, 20, and 30 min of base immersion. Sample weights were then measured each one hour since the beginning of sample base immersion.

The weights of samples at each of the specified times are measured as discussed earlier, and the absorption of sample is calculated using Equation (1).

(1) $$I=\frac{m_{t}}{a\times d}$$

where:

I= absorption

$m_{t}=$ the change in specimen mass in grams, at the time *t*

a= the exposed area of the specimen, in mm^2^, and

d= the density of water, taken as 0.001 g/mm^3^

A common model for the relationship between the absorption and the square root of time for the first six hours of absorption measurement is given by Equation (2) [ASTM C1585]. The linearity of the relationship between the water absorption “*I*” and the square root of time was validated in several studies [34].

(2) $$I=S_{i}\sqrt{t}+b$$

where:

$S_{i}=$ initial rate of absorption, mm/$\sqrt{s}$.

### 2.3. Experimental Procedure–Resistance to Sulfuric Acid Attack

Response of alkali-activated mortar samples to sulfuric acid exposure was assessed using ASTM C267 [37] test procedure. 20 mm cubic samples were prepared and left in molds for 24 h, then demolded and weighed using 0.01 kg scale. Samples were then submerged in 10% sulfuric acid (H_2_SO_4_) until the test day. After 7-days, 28-days, and 90-days of curing, samples were surface-dried, weighed, and the change in weight was determined. Sufficient specimens were cast so the results were the average of three samples for each target parameter including activator ratio, curing age, molarity, and binder combination. The percentage change in sample weight was calculated using Equation (3).

(3) $$weight\;change\left(\%\right)=\left[\left(W-C\right)/C\right]\times 100$$

where:

*C* = conditioned weight of the specimen (grams)

*W* = weight of specimen after immersion (grams)

### 2.4. Initial and Final Setting Times

One of the goals of this study is to evaluate the effects of two factors on the initial and final setting times: (1) the relative amounts of GGBS and fly ash in the total binder content, and (2) the molarity of sodium hydroxide (NaOH) activator. The setting time of the mixes described earlier was determined using ASTM C191 and calculated Vicat time (minutes) is given by:

$$\left(\left(\frac{H-E}{C-D}\right)\times \left(C-25\right)\right)+E$$

where:

*E* = time in minutes of the last penetration greater than 25 mm.

*H* = time in minutes of the first penetration less than 25 mm.

*C* = penetration reading at time *E*, and

*D* = penetration reading at time *H*.

## 3. Results and Discussion

### 3.1. Sorptivity and Mass Loss

**Figure 2:**
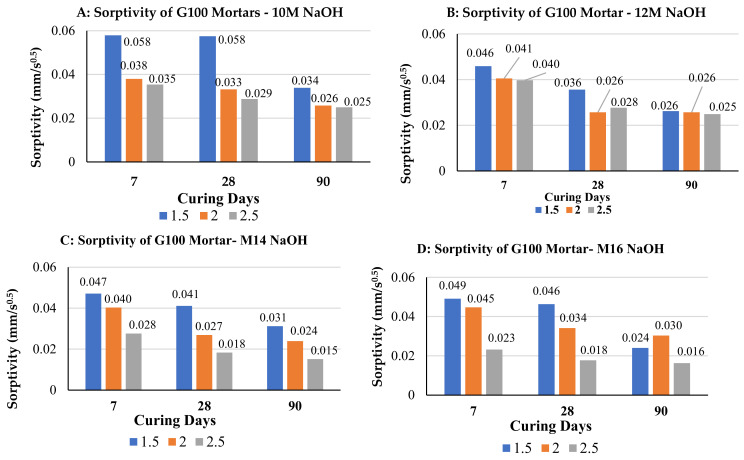
Effect of Na_2_SiO_3_/NaOH ratio sorptivity of 100%GGBS alkali activated mortar specimens when molarity of NaOH is: (**A**) 10 M, (**B**) 12 M, (**C**) 14 M, (**D**) 16 M.

Figure 2 shows the calculated initial rate of absorption $s_{i}$ (mm/$\sqrt{s}$) for G100 mortar samples activated with sodium hydroxide and sodium silicates solution combined so that the alkaline activator ratio Na_2_SiO_3_/NaOH is 1.5, 2.0, or 2.5. Mortar sample were created with these three activator ratios for each of four NaOH molarities: 10 M, 12 M, 14 M, and 16 M. In general, the higher the ratio of Na_2_SiO_3_/NaOH the lower the sorptivity of mortar samples, especially for higher molarities of the NaOH activator (14 M and 16 M). It is also clear that for each activator ratio (1.5, 2.0, or 2.5), sorptivity decreases with curing age and the phenomenon is true for all of the four NaOH moralities. This is because more hydration and polymerization products develop over time which results is filling more voids. It was reported that in slag-fly ash blends, where slag content is greater than or equal 50%, spacing-filling calcium (alumina) silicate hydrate gel provides porosity reductions not observed in sodium aluminosilicate geopolymer gels [38].

**Figure 3:**
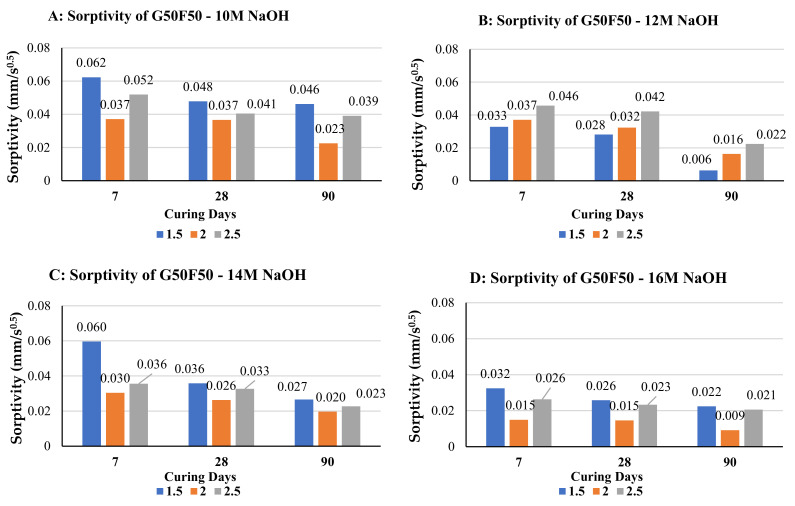
Effect of Na_2_SiO_3_/NaOH ratio Sorptivity of 50%GGBS+50%fly ash alkali activated mortar specimens when molarity of NaOH is: (**A**) 10 M, (**B**) 12 M, (**C**) 14 M, (**D**) 16 M.

Figure 3 shows the influence of Na_2_SiO_3_/NaOH ratio on sorptivity of alkali-activated G50F50 mortar samples. Figure 3A–D represent sorptivity of samples prepared with NaOH molarity of 10 M, 12 M, 14 M, and 16 M respectively. Similar to the pattern with G100, sorptivity decreases with curing age, for each NaOH molarity and for each Na_2_SiO_3_/NaOH ratio considered in this study. Mortar samples prepared with Na_2_SiO_3_/NaOH ratio of 2.0 experienced the lowest sorptivity when tested after each of the three curing ages (7, 28, and 90 days) for samples prepared with NaOH molarities of 10 M, 14 M, and 16 M. This ratio appears to represent the optimum sodium to produce polymerization products such as calcium sodium aluminosilicate hydrate (C-N-A-S-H) from the fly ash precursor, which represents 50% of the total binder. At the age of 28-days and alkaline activator ratio of 2.0, sorptivity ranged from 3.66 $\mu mm/\sqrt{s}$ when NaOH molarity was 10 M, to 1.46 $\mu mm/\sqrt{s}$ when NaOH molarity increased to 16 M. Hydration of the other 50% GGBS continues and produces products such as calcium aluminosilicate hydrate (C-A-S-H) and decreases the voids. The decrease in sorptivity with increase in alkaline activator molarity of G50F50 will be shown in subsequent sections of this article to be consistent with the increase in compressive strength with increase in solution alkalinity.

**Figure 4:**
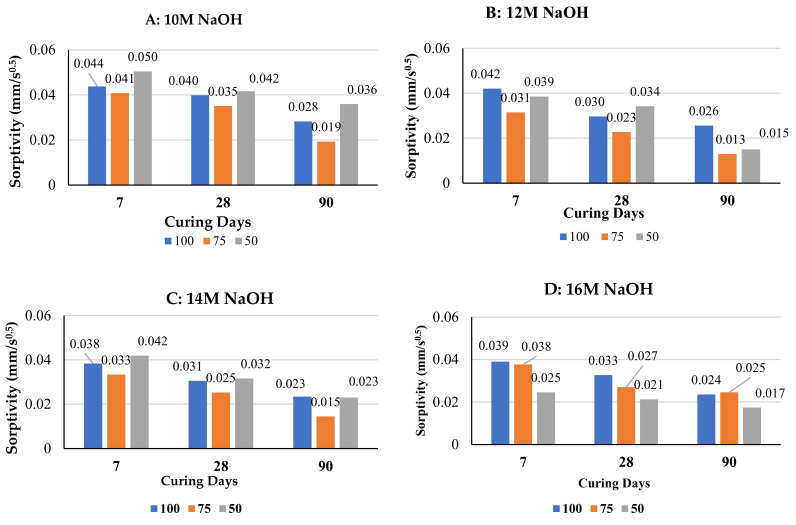
Change of sorptivity with curing age of 100%GGBS, 75%GGB+25%FA, and 50%GGB+50%FA, for molarity of NaOH of: (**A**) 10 M, (**B**) 12 M, (**C**) 14 M, and (**D**) 16 M.

The variation of sorptivity rate with curing age for the three binder types/combinations including G100, G75F25, and G50F50 is shown in Figure 4 for NaOH molarities of 10 M, 12 M, 14 M, and 16 M. The lowest sorptivity after 7 days, 28 days, or 90 days of curing is exhibited by G75F25 for NaOH molarities of 10 M, 12 M, and 14 M. For NaOH molarity of 16 M, G50F50 mortars developed the lowest sorptivity after 7, 28, and 90 days of curing compared to the other two binder combinations. For all NaOH molarities and for each of the three binder combinations, sorptivity decreased with increase in sample curing age. This due to the formation of more hydration and polymerization products. Similarly, increasing fly ash content from 25% to 50% increased sorptivity after each curing age of 7, 28, and 90 days and for molarities of NaOH of 10 M, 12 M, and 14 M. This is consistent with the findings of Mehta [39] for mortar samples tested at the age of 28 days, where increasing GGBS content up to 20% in fly ash-GGBS blends for a particular molarity decreased sorptivity. This is because of the slow dissolution of fly ash and formation of polymerization products at ambient temperature which delays of filling of voids when fly ash content is high. Curing temperature generally affects both hydration of OPC and polymerization of fly ash, therefore, influences rate of strength development [40,41].

### 3.2. Effect of Exposure to Sulfuric Acid on Mass of Alkali-Aactivated GGBS-Fly Ash Mortar

**Figure 5:**
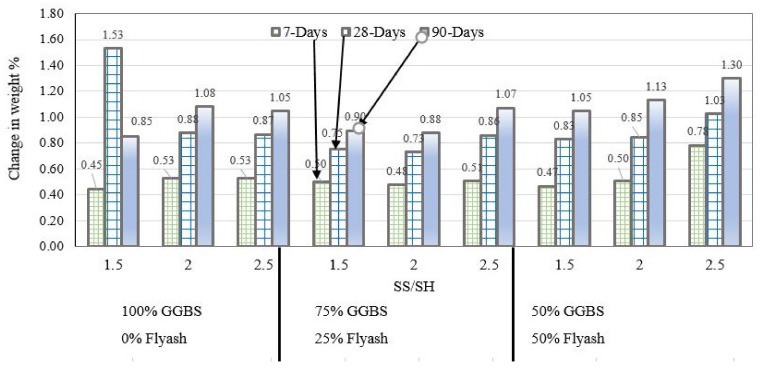
Weight change due to submission of mortars in 10% sulfuric acid for G100, G75F25, and G50F50 binders.

As discussed in the introduction of this paper, extended exposure of concrete to acid may lead to loss of mass and strength. Figure 5 shows the change in sample weight due to submersion of mortar samples in 10% sulfuric solution. In general, mortar samples weight increased with increase in activator ratio from 1.5 to 2.5 and the pattern was consistent for each of the three binder combinations G100, G75F25, and G50F50. Therefore, sulfate attack did not cause loss of mass over time, instead, curing the samples under water caused increase in weight due to formation of geopolymerization and hydration products. The formation of geopolyermization and hydration products outpaced any decalcification or dealumination that may have been caused by acid attack, largely due to the co-existence of fly ash and GGBS in quantity greater than 50%. In contrast, it was reported in the literature that when fly ash is used as sole binder in alkali-activated mortars, loss of mass may occur as early as 14 days after immersion in acid solution [19]. As discussed in the previous sections, sorptivity decreased with time for all binder combinations and alkaline activator concentrations, which is consistent with increase in mass reported in this section. Similarly, in subsequent sections of this paper, it will be shown that compressive strength also increases with time.

### 3.3. Effect of NaOH Molarity on Initial Setting (IS) and Final Setting (FS) Times

Initial setting (IS) and final setting (FS) times are critical fresh properties for successful use of concrete with alkali-activated GGBS and fly ash binders. The effect of molarity of NaOH and relative contents of GGBS/fly ash in binder are discussed in this section.

**Figure 6:**
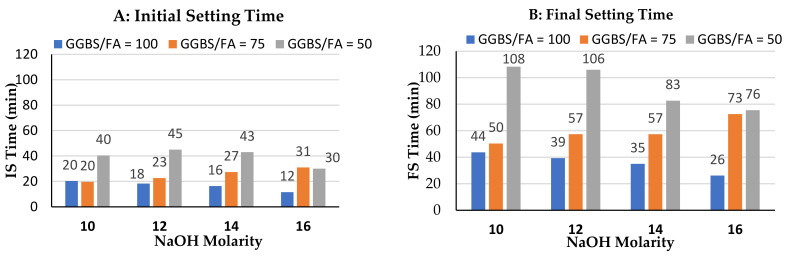
Variation of setting time with molarity of NaOH activator for 100% GGBS binder, 75%GGBS+25%FA binder, and 50%GGBS+50%FA binder (**A**) initial setting time (IST), (**B**) final setting time.

Figure 6 shows the initial and final setting times for each of the three binder combinations and 4 values of NaOH molarity. G100 mortar samples exhibited decrease in both initial and final setting times with increase in molarity of the NaOH activator. The initial setting time was very short ranging from 20.33 min for 10 M NaOH to 11.5 min for 16 M NaOH. The final setting time was also short ranging from 43.67 min for 10 M NaOH to 26.17 min for 16 M NaOH. Increasing the molarity of NaOH for G100 mortar samples increases the supply of calcium and formation of calcium silicates gel, which shortens both initial and final setting times. Calcium is abundant in GGBS precursor in the form of CaO. The fast dissolution of Al and Si, in addition to abundance of calcium, modifies the network and forms reaction products at faster rate [29]. 

G75F25 mortars exhibited an increase in initial and final setting times with increase in molarity of the activator NaOH, except for 16 M where setting time decreased compared 14 M. The presence of 25% fly ash in the binder increases both initial and final setting time compared to 100%GGBS binder for each value of the NaOH molarities. This is due to the inherently slow polymerization of fly ash. Initial setting time for G75F25 ranges from 19.67 min to 31 min, depending on NaOH concentration. 

The effect of higher amounts of GGBS in alkali-activated GGBS/fly ash is to decrease the setting time as shown in Figure 6A,B, which is consistent with the literature [27]. The fast setting time associated with the presence of GGBS may be addressed in various ways, such as the addition of up to 2% microsilica [29]. Borax may also be used to increase setting time [42].

When the amount of fly ash is increased to 50% of the total binder, the initial and final setting times increase significantly as a results of longer time needed to polymerize a larger amount of fly ash in the binder. Initial setting time of G50F50 mortar ranged from 30 min to 45 min, while the final setting time ranged from 75.5 min to 108.3 min. 

In general, the setting time of all mixes made with these three binders is relatively short compared to 100% fly ash geopolymer concrete and compared to OPC-based concrete. The short setting time of all three mixes, at room temperature is due to the presence of GGBS which hydrates and releases calcium into the system, and speeds up the formation of calcium silicates gel. In addition, it was shown that the higher the initial ratio of SiO_2_/Al_2_O_3_ in the mix, the lower the initial setting time, which is the case in binders with higher GGBS contents [26]. 

Figure 6B shows that the final setting for G100 mortars, decreases with increase in the molarity of NaOH. Once 25% fly ash is added (G75F25), final setting time increases compared to G100 mortars, but the trend remains that setting time increases with molarity of activator NaOH. When the percentage of fly ash is further increased to 50% (G50F50), setting time increases further compared to G100 and G75F25, but the trend now reverses so that an increase in molarity decreases the setting. This latter trend is consistent with the findings of Lee and Lee [43] although, in that study, the molarity of NaOH was increased from 4 M to 8 M and the fly ash to GGBS ratio was 4:1.

### 3.4. Effect of 10% Sulfuric Acid Solution on Compressive Strength Development

To observe the effect of sulfuric acid on compressive strength development, samples were tested in compression at the ages of 7-days, 28-days, and 90-days. As discussed earlier, mortar samples were removed from moulds 24 h after casting, and were then placed in 10% sulfuric acid until compression test day.

Table 3 shows the compressive strength of the cubic mortar samples prepared using 100% GGBS, 75%GGBS + 25% fly ash, and 50% GGBS + 50% fly ash. Each compressive strength value in the table is a function of binder type, alkaline activator ratio sodium silicate/sodium hydroxide (SS/SH), and molarity of NaOH alkaline activator. 

After 7 days of curing under 10% sulfuric acid, the highest compressive strength of 56.96 MPa was developed by the G75F25 mix when the molarity of NaOH was 10 M and SS/SH = 1.5, although G100 developed a statistically similar compressive strength of 56.66 MPa at the same NaOH molarity and SS/SH ratio. This is due to the fast dissolution of GGBS in the early days after, compared to the slower polymerization of fly ash, especially at ambient temperature of 22±2 0C in which the samples in this study were cured. This is consistent with published literature on reaction products of 80%/20% GGBS/fly ash blends where C-A-S-H was detected after one day of mixing and attributed to the abundance of Ca2+ ions that are readily available to react with Si and Al ions leading to precipitation of C-A-S-H gel, while no N-A-S-H was found at such early age [31]. 

After 28-days of curing, G75F25 continued to develop the highest compressive strength of 80.53 MPa compared to G100 and G50F50 mixes. This indicates GGBS not only continued to hydrate at a fast rate, but also accelerated the polymerization of the 25% fly ash in G75F25 mixes leading to increase in hydration and polyermization products. For this optimum combination of 75%GGBS and 25% fly ash to increase compressive strength to its highest value at the age of 28-days, NaOH molarity needed to increase to 12M. The high 28-day compressive strength, even with relatively high liquid-to-binder ratio of 0.55 is attributed to the optimum compactness of the system consisting of dense less porous C-A-S-H due to the high content of GGBS, combined with smaller amount of the more porous hardened matrix of N-A-S-H. The larger permeable voids of N-A-S-H compared to the denser and smaller pore system of C-A-S-H is extensively documented in the literature [30,37].

After 90 days of curing, G75F25 continued to develop the highest compressive strength of 91.06 MPa, compared to the other two binders. The concentration of activator NaOH was the lowest of 10 M or 12 M when G75F25 reached the highest compressive strength after 90 days, due to the relatively small amount of fly ash. However, G50F50, which contains the highest fly ash content in this study, developed 86.73 MPa after 90 days of curing when NaOH concentration was 16 M, which is very close in magnitude to the highest strength achieved by G75F25 during the same period of time. This was the case because the high alkalinity of the solution facilitated polymerization of larger amounts of the residual 50% fly ash during the 90 days of submersion under water. The dissolution of fly ash contributed higher amounts of silicates and aluminates, along with the higher supply of sodium ions through NaOH led to formation of N-A-S-H and caused the observed significant increase in compressive strength. The effect of high solution alkalinity on compressive strength, in relation to the content of fly ash is further evidenced after 90 days by comparing the strengths of G100, G75, and G50F50. The least compressive strength after 90 days with 16 M NaOH was developed by G100 mortar, while the highest was developed by either G75F25 or G50F50, each of them contains higher content of fly ash compared to G100. However, it is documented that the pore system produced in GGBS-fly ash blends when GGBS content is high, is denser and more compact, compared to the matrix produced with high fly ash content [27]. It may therefore be hypothesized that while the pore structure of the matrix formed by polymerization of fly ash is coarse and permeable, when fly ash content less than or equal to 50%, it still contributes significantly to long-term compressive strength development when samples are water cured. This is possibly due to the effective filling of the porous pore system contributed by fly ash with the finer and denser pores developed by GGBS. 

When cured in water under ambient temperature, lower NaOH concentration (10 M and 12 M) enhances the strength development when fly ash content is low (less than 50% in this study), while higher NaOH concentration (16 M in this study) contributes to higher strength, in the long term when fly ash content is relatively high (50% in this study).

**Figure 7:**
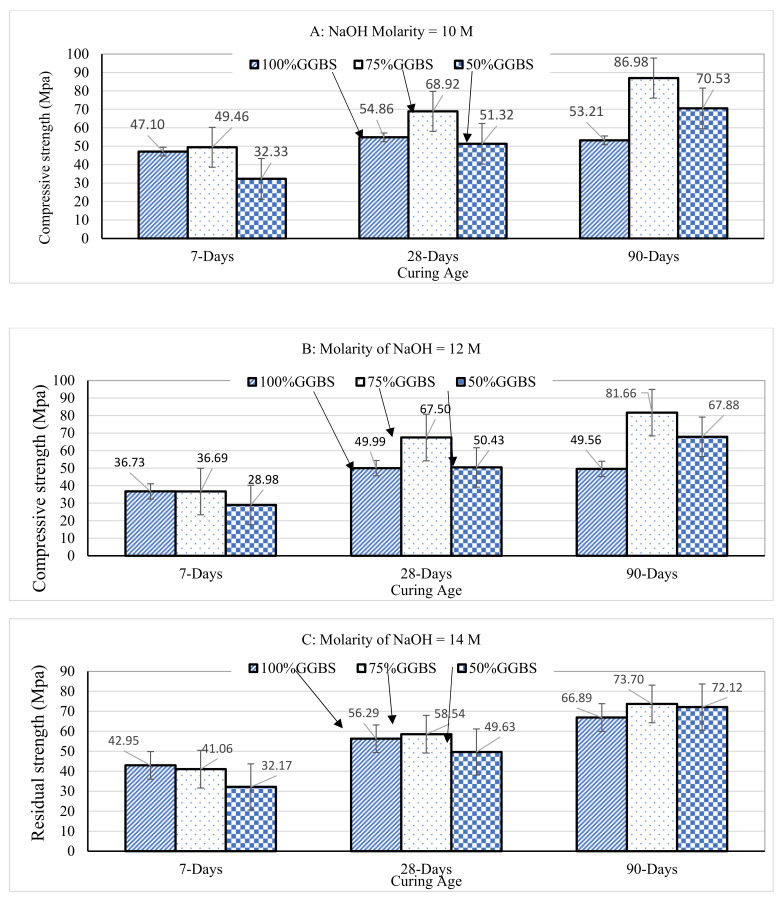

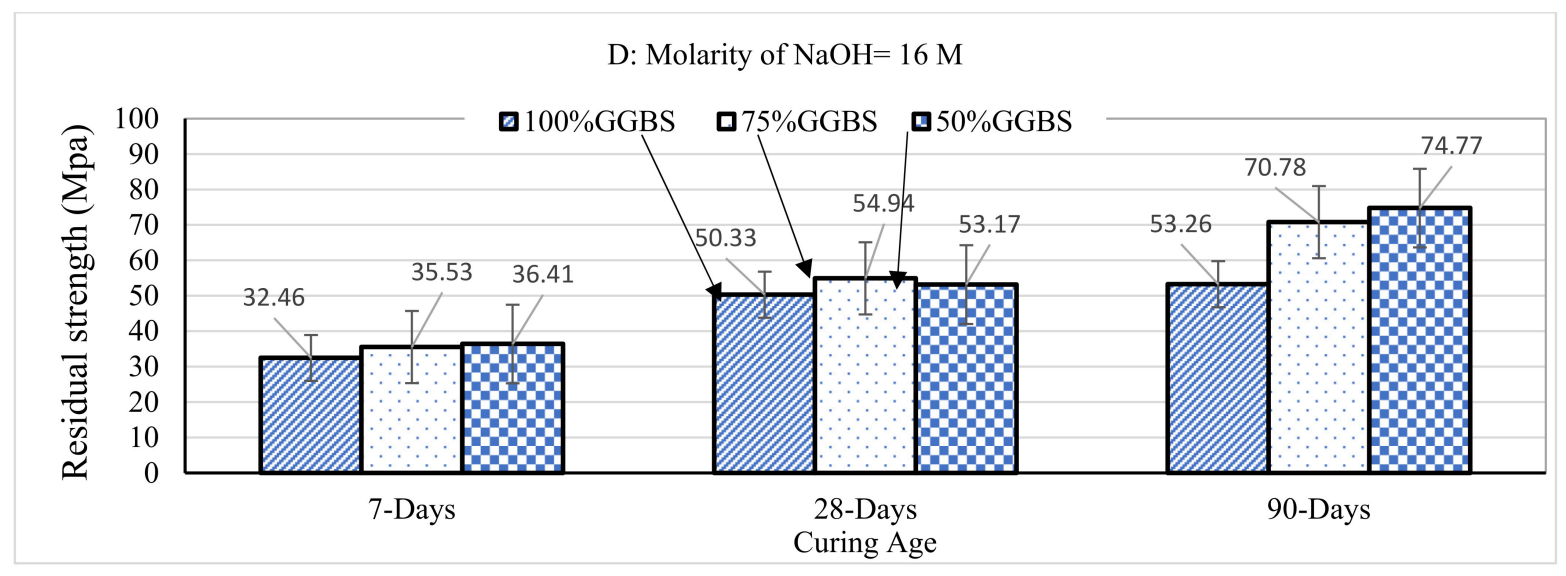
Compressive strength development for samples submerged under 10% sulfuric acid until compression test day. (**A**): Molarity of NaOH activator is 10 M, (**B**): Molarity of NaOH activator is 12 M, (**C**): Molarity of NaOH activator is 14 M, and (**D**): Molarity of NaOH activator is 16 M.

It is clear now that submersion of mortar samples that use GGBS-fly ash blends as binder in 10% sulfuric acid solution for up to 90 days doesn’t cause reduction in mass nor compressive strength. Fly ash matrix is typically more susceptible to damage due to sulfuric acid, but in the present study it is limited to 50% of the total binder content. We now examine the effect of NaOH concentration, and binder composition on compressive strength development. Figure 7A–D, show the development of compressive strength with curing age for NaOH molarities of 10 M, 12 M, 14 M, and 16 M. 

After 7 days of curing, mortar samples with the largest concentration of GGBS, such as G100 developed the highest compressive strength, due to the fast dissolution of GGBS compared to fly ash. As shown in Figure 7A–C, G100 developed higher or similar 7-day compressive strength as G75F25 mortars for all concentrations of the activator NaOH, while the strength of G50F50 mortars was relatively low, dominated by the slow polymerization of fly ash. However, Figure 7D, corresponding to NaOH molarity of 16 M, shows that G75F25 and G50F50 developed similar 7-day compressive strength, that is slightly higher than the G100 mix. This due to the higher supply of sodium from the 16 M NaOH forming sodium-aluminate-silicate hydrates, although at slow rate.

As reaction and polymerization continue to 28-days, G75F25 mortar samples developed higher compressive strength than G100 and G50F50. As shown in Figure 7A, the average 28-day compressive of G75F25 mortars was nearly 69 MPa, the highest of the three binder combinations which certainly classifies as high strength concrete for most practical applications. Nonetheless, G50F50, affected by low dissolution of fly ash still developed 28-day of 51.33 MPa, also high strength for structural engineering applications. This is different from the data published by Hu [38] which showed that 28-day compressive strength to increases with increase in GGBS content, without an optimum GGBS content. The 10 M molarity is the optimum NaOH concentration for 28-day compressive strength development for the G75F25 binder. Figure 7A–C shows that G100 mortars decreased in compressive strength at the age of 28 days, below G75F25, regardless of the alkaline activator concentration, but remain higher than or equal to G50F50 samples, indicating larger percentage of GGBS was consumed through formation of hydration products for NaOH concentrations of 10 M, 12 M, and 14 M. At NaOH concentration of 16 M, the 28-day compressive strength of G50F50 reaches that of G75F25, but exceeds G100, similar to the 7-day situation discussed in the previous section.

After 90 days of curing, G75F25 continued to dominate compressive strength performance of the three binders, reaching an impressive average strength of 87 MPa as shown in Figure 7A. This confirms NaOH concentration of 10 M is optimum for compressive strength development of GGBS-fly ash binders and alkaline activator concentrations covered in this study. After 90 days of curing, G50F50 mortars reached compressive strength of 74.7 MPa, surpassing the compressive strength of G75F25 as well as G100 for NaOH concentration of 16 M, as shown in Figure 7D. Recall that the strength of G75F25 was essentially equal to G50F50 samples at 16 M concentration after 7-days and 28-days of curing. The slow dissolution of fly ash delayed the strength development of G50F50, but the abundance of sodium supplied by the higher concentration of NaOH at 16 M molarity, led to formation of additional sodium-based silicate gel at the age of 90 days. This led to G50F50 surpassing the strength of G100 and G75F25, for this particular NaOH concentration.

Regardless of the NaOH concentration, the strength development rate for G50F50 is higher from the age of 28 days to the age of 90 days, compared to the rate from 7-days to 28 days. This is particularly true for higher concentrations of 14 M and 16M where the strength of G50F50, clearly approaches, then exceeds the strength of G75F25, as shown in Figure 7C,D. The higher alkalinity environment supports the continuity of fly ash dissolution with time, and the abundant supply of sodium to keep strength development continuing.

## 4. Conclusions

This study evaluated selected factors influencing sorptivity, setting time, and sulfuric acid resistance, of alkali-activated mortar samples in which the binder is either GGBS or combination or GGBS and fly ash. Effect of submersion in 10% sulfuric acid solution for up to 90 days on compressive strength development was also evaluated. These factors include the relative amounts of GGBS and fly ash in the binder, the ratio of sodium silicate-to-sodium hydroxide, and molarity of the sodium hydroxide alkaline activator. The findings apply to alkaline activator consisting of sodium silicate and sodium hydroxide with Na_2_SiO_3_/NaOH ranging from 1.5 to 2.5. The concentration of the alkaline activator was adjusted by varying NaOH concentration from 10 M to 16 M in increments of 2 M. The following observations were made:

- Sorptivity decreases with curing age, regardless of the molarity of NaOH activator and regardless of the ratio of sodium silicate/sodium hydroxide. This pattern remains consistent for each binder composition, including 100%GGBS, 75%GGBS+25% fly ash, and 50%GGBS + 50%fly ash. The optimum binder combination/activator mix, undergoing the lowest sorptivity and potentially enhancing durability, contains 75%GGBS + 25% fly ash with NaOH activator molarity of 12 M. The lowest sorptivity was exhibited by this combination after each of the three tested curing ages (7, 28, and 90 days), compared to the other two binder combinations (100%GGBS and 50%GGBS+50%fly ash). This indicates formation of hydration and polymerization products leading to filling of voids is optimum at this binder combination.
- For the mix with 100% GGBS binder, sorptivity tends to decrease with increase in the ratio of sodium silicate/sodium hydroxide, when NaOH = 10 M, 12 M, 14 M, and 16 M. Samples prepared with binder combination 50%GGBS+50% fly ash experienced the lowest sorptivity after each curing age (7, 28, and 90 days) when the activator ratio is 2.0 compared to samples prepared using activator ratios of 1.5 and 2.5.
- Alkali-activated mortar samples prepared using alkali-activated GGBS/fly ash as binders did not experience visible damage nor mass loss when subjected to sulfuric acid for up to 90 days. To the contrary, mass of mortar samples increased after submersion in sulfuric acid for all GGBS/fly ash binder combinations. This is because the maximum fly ash content in the three binder combinations evaluated in this study was 50%.
- Compressive strength of mortar samples submerged in 10% sulfuric acid solution increased with age, for all binder combinations and all concentrations of NaOH activator. While sulfuric may have affected the matrix, hydration and polymerization products clearly supplemented and enhanced the pore system decreasing sorptivity and increasing compressive strength. The observation however, is limited to the curing in water for a maximum period of 90-days, and fly ash limit of 50% of total binder content.
- Mortars with binder consisting of 75%GGBS+25% fly ash developed the highest compressive strength after 7-days, 28-days, and 90-days of curing, compared to 100%GGBS and 50%GGBS+50%fly ash binder. This binder combination was also the optimum to produce minimum sorptivity in all curing ages. The optimum NaOH concentration for 75%GGBS+25% fly ash binder to develop the highest 28-day compressive strength and lowest sorptivity is 12 M.
- The 50%GGBS+50%fly ash mix developed a high 90-day compressive strength of 86.74 MPa when NaOH concentration was 16 M, corresponding to the highest solution alkalinity in this study.
- Initial and final setting times of mortar samples increase with molarity of NaOH when the binder is 100%GGBS and when the binder consists of 75%GGBS+25%fly ash. Similarly, setting time of mortar samples increases with increase in fly ash content from 0% to 50%.

## Figures and Tables

**Table 1:** Properties of ground granulated blast furnace slag and fly ash.

	CaO	SiO_2_	Al_2_O_3_	SO_3_	Fe_2_O_3_	TiO_2_	K_2_O	MnO	SrO	ZrO_2_	CuO	Cr_2_O_3_	Y_2_O_3_
GGBS (%)	59.44	25.68	8.12	2.75	1.499	1.048	0.609	0.562	0.125	0.065	0.031	0.026	0.016
Fly Ash (%)	4.294	58.158	24.351	0.068	8.517	2.565	1.534	0.094	0.062	0.085	0.037	0.047	0.015

**Table 2:** Mortar mixes tested in the program, based on binder type, molarity of NaOH, and ratio of Sodium Silicate/Sodium Hydroxide.

	G100 Mixes				G75F25 Mixes				G50F50 Mixes			
	GGBS (%)	Fly Ash (%)	NaOH Molarity (M)	SS/SH	GGBs (%)	Fly Ash (%)	NaOH Molarity (M)	SS/SH	GGBs (%)	Fly Ash (%)	NaOH Molarity (M)	SS/SH
1	100	0	10	1.5	75	25	10	1.5	50	50	10	1.5
2	100	0	10	2	75	25	10	2	50	50	10	2
3	100	0	10	2.5	75	25	10	2.5	50	50	10	2.5
4	100	0	12	1.5	75	25	12	1.5	50	50	12	1.5
5	100	0	12	2	75	25	12	2	50	50	12	2
6	100	0	12	2.5	75	25	12	2.5	50	50	12	2.5
7	100	0	14	1.5	75	25	14	1.5	50	50	14	1.5
8	100	0	14	2	75	25	14	2	50	50	14	2
9	100	0	14	2.5	75	25	14	2.5	50	50	14	2.5
10	100	0	16	1.5	75	25	16	1.5	50	50	16	1.5
11	100	0	16	2	75	25	16	2	50	50	16	2
12	100	0	16	2.5	75	25	16	2.5	50	50	16	2.5

**Table 3:** Compressive strength development of mortar samples cured in 10% sulfuric acid solution for G100, G75F25, G50F50 for various concentration of NaOH and three ratios of sodium silicate/sodium hydroxide.

		7 Days			28 Days			90 Days		
NaOH Molarity	SS/SH	G100	G75F25	G50F50	G100	G75F25	G50F50	G100	G75F25	G50F50
10 M	1.5	56.66	56.96	43.66	66.86	61.83	55.43	74.56	81.66	74.6
10 M	2	39.53	40.96	26.5	61.2	75	53.23	45.63	91.06	70.86
10 M	2.5	45.1	50.46	26.83	36.53	69.93	45.3	39.43	88.23	66.13
12 M	1.5	22	35.5	29.23	47.26	65.13	44.93	37.03	73.9	66.66
12 M	2	31.9	34.63	29.3	40.7	56.83	52.96	48.93	84.03	67.63
12 M	2.5	56.3	39.93	28.4	62	80.53	53.4	62.73	87.06	69.36
14 M	1.5	35.76	39.16	26.76	53.3	45.96	40.76	54.16	60.96	62.7
14 M	2	43.5	44.73	33.03	56.63	60.53	51.9	73	80.23	73.83
14 M	2.5	49.6	39.3	36.73	58.93	69.13	56.23	73.5	79.9	79.83
16 M	1.5	22.63	19.13	27.63	36.93	38	38.06	37.96	51.26	63.53
16 M	2	42.43	47.76	44.06	50.93	65.56	69.2	51.8	78.06	86.73
16 M	2.5	32.33	39.7	37.53	63.13	61.26	52.26	70.03	83.03	74.06

## Data Availability

All necessary data provided in the article.
